# Twin and triplet discordance in retinopathy of prematurity: a call to integrate genomic, placental, and environmental determinants

**DOI:** 10.1186/s40942-025-00714-0

**Published:** 2025-07-28

**Authors:** Zahra Sharifi, Mojtaba Heydari, Mohammadkarim Johari, Shayan Yousufzai

**Affiliations:** 1 Department of Master of Public Health, Shiraz University of Medical Sciences, Shiraz, Iran; 2 Poostchi Ophthalmology Research Center, Department of Ophthalmology, School of Medicine, Shiraz University of Medical Sciences, Shiraz, Iran; 3 Medical Education Department, Education Development Center, Shiraz University of Medical Sciences, Shiraz, Iran

**Keywords:** Retinopathy of prematurity (ROP), Multiple births, Twins, Triplets, ROP discordance

## Abstract

**Background:**

Retinopathy of prematurity (ROP) is a major cause of preventable blindness in preterm infants, especially in multiple births such as twins and triplets. Although the incidence of ROP in these groups is well-documented, the discordance in disease severity among siblings has not been thoroughly investigated. This study aimed to quantify the discordance of ROP in twins and triplets and to identify associated predictive factors.

**Methods:**

A cross-sectional study was conducted at the Poostchi ROP Clinic in southern Iran, involving 339 preterm infants from twin and triplet pregnancies undergoing screening for ROP. Data were collected on demographic, clinical, and ROP-specific variables, including gestational age (GA), birth weight (BW), duration of oxygen therapy, and ROP stage. Discordance was defined as a difference in ROP stage between siblings. Stepwise Poisson regression was employed to identify predictors of discordance.

**Results:**

ROP discordance was observed in 88 neonates (26.0%), comprising 52 twins and 36 triplets. GA emerged as the only significant predictor of ROP severity (prevalence ratio = 0.90, 95% CI: 0.83–0.97, *p* = 0.012). No other variables, including BW, sex, or medical interventions, demonstrated significant associations. A post-hoc power analysis revealed limited statistical power for detecting subtle effects or rare events, indicating the necessity for larger studies.

**Conclusion:**

ROP discordance was present in over one-quarter of neonates from multiple births, with GA as the primary influencing factor. The multifactorial nature of discordance highlights the need for larger, multicenter studies that incorporate genetic, placental, and prenatal data to optimize individualized neonatal care and prevent vision loss in this high-risk population.

## Introduction

Retinopathy of prematurity (ROP) is a critical vascular disorder affecting premature infants, potentially leading to severe visual impairment or blindness if untreated. Globally, approximately 184,700 preterm infants develop ROP annually, with 20,000 progressing to blindness or severe visual impairment [1]. The prevalence of ROP varies by region and population, affecting 20–30% of infants born before 32 weeks gestational age (GA) or weighing less than 1500 g in high-income countries, and up to 35–40% in low- and middle-income countries, with rates as high as 68% in very low birth weight infants (< 1251 g) (Early Treatment for Retinopathy of prematurity cooperative group, 2003). In settings like India, ROP incidence ranges from 20 to 51% in infants born at less than 34 weeks or weighing under 1750 g [2]. These figures highlight the significant burden of ROP and the urgent need for effective screening and intervention strategies to mitigate its impact on neonatal vision.

Multiple pregnancies, such as twins and triplets, are inherently associated with higher risks of prematurity and low birth weight, key predisposing factors for ROP. Studies indicate that ROP prevalence in multiples ranges from 12 to 54%, potentially higher than in singletons, though differences often diminish when adjusted for GA and birth weight (BW) [3, 4]. For instance, a Brazilian study reported a 53.6% incidence of any stage ROP in twins and triplets compared to 35% in singletons, though severe (threshold) ROP rates were similar [4] Higher-order multiples, such as quadruplets, may exhibit elevated ROP rates, with one study noting a 60% prevalence [3]. These findings underscore the complex interplay of multiple gestation and ROP risk, necessitating further investigation into specific patterns within these populations.

While ROP is not uncommon in twins and triplets, the discordance in ROP severity—where siblings within the same pregnancy exhibit different stages of the disease—remains a relatively understudied phenomenon. This discordance is particularly intriguing because twins and triplets share similar genetic backgrounds and intrauterine environments. However, variations in factors such as birth weight, placental sharing, and postnatal care may lead to divergent ROP outcomes. The existing literature on ROP discordance is limited, with most studies concentrating on the overall prevalence or severity of ROP in multiples rather than identifying the specific predictors of discordance.

For example, a study on discordant twins found that lower BW twins had a higher risk of ROP, but this association weakened after adjusting for GA [5]. Similarly, a case report on triplets highlighted the variable ROP severity of ROP and the differing treatment needs among siblings, suggesting that unexamined factors, such as weight gain or placental characteristics, may play a role [6]. This lack of research on discordance, particularly in triplet births, represents a significant gap in our understanding of the mechanisms driving ROP variability in ROP among multiple births.

To address this critical gap, our study investigates the discordance of ROP in twins and triplets. We aim to quantify its prevalence and identify predictive variables that contribute to differences in ROP severity among siblings.

## Methods

### Study design, setting, and objectives

This cross-sectional study was conducted at the Poostchi ROP Clinic (affiliated with Shiraz University of Medical Sciences, southern Iran), a specialized tertiary care center for ROP screening, diagnosis, and management following the ethical approval under reference ID “IR.SUMS.REC.1402.476. This study was conducted in accordance with the ethical standards laid down in the Declaration of Helsinki and was approved by the institutional ethics committee. Informed consent was obtained from the parents or legal guardians of all participants. The study was conducted retrospectively over a period from January 2020 to February 2024 based on medical records of preterm infants who underwent routine ROP screening during this timeframe.

The primary objective was to investigate discordance in ROP severity and progression among preterm infants from multiple pregnancies (twins and triplets), identifying distinct risk factors influencing its development. Secondary aims were to quantify the prevalence of ROP discordance and explore potential predictors to inform tailored neonatal care strategies; hence, the first ophthalmological examination was performed at 31 weeks postmenstrual age or 4 weeks after birth, whichever came later, in accordance with established screening guidelines. Subsequent examinations were scheduled based on the findings of the initial screening, with follow-up intervals determined by the stage and zone of ROP observed, in line with International Classification of Retinopathy of Prematurity (ICROP) recommendations and national screening protocols.

### Eligibility criteria

Preterm infants from multiple pregnancies (twins and triplets) undergoing routine ROP screening per southern Iran national guidelines were eligible. These guidelines mandate screening for neonates born at ≤ 34 weeks GA or with BW < 2000 g. Neonates exceeding these GA/BW thresholds were included if referred due to unstable health status in the neonatal period (e.g., respiratory distress, hemodynamic instability, suspected infection requiring intensive monitoring). Exclusion criteria encompassed infants with congenital ocular anomalies, genetic syndromes predisposing to ocular complications, incomplete/inadequate ROP screening, or insufficient follow-up data.

### Data collection and variables

Data were collected by trained general physicians, Master of Public Health students, and epidemiologists under the supervision of an attending ophthalmologist, following a standardized protocol to ensure quality. Documented variables included:

- **Demographic/Clinical**: BW, GA, oxygen saturation levels, sex, maternal age, method of delivery, number of siblings (twins/triplets), number of blood transfusion(s), presence of sepsis, presence of systemic disease (any neonatal comorbidities, including the following categories: pulmonary, cardiovascular, neurological, gastrointestinal, thyroid, renal, hematologic, and endocrine disorders), length of NICU stay in days, duration of mechanical ventilation in days, duration of oxygen therapy when extubated in days.
- ROP-Specific Variables: Data were collected on the stage of retinopathy, inter-sibling stage differences, and the need for treatment including laser photocoagulation or intravitreal bevacizumab (IVB) therapy. Staging of ROP and classification of discordance were based on ICROP criteria following as: **Mild**: One-stage difference (e.g., Stage 1 vs. Stage 2), **Moderate**: Two-stage difference (e.g., Stage 1 vs. Stage 3), and **Severe**: Three-stage or greater difference, or a discrepancy where one sibling required treatment (laser or intravitreal bevacizumab) while the other did not [5, 11].

### Statistical analysis

Descriptive statistics characterized variable distributions, reporting means and 95% confidence intervals for continuous variables (GA, BW, O2 duration, NICU stay, ventilation duration, maternal age) and frequencies for categorical variables (ROP stages, treatments, discordance prevalence). Normality testing was conducted solely for GA, utilizing the Shapiro-Wilk and Kolmogorov-Smirnov tests. These tests were not performed for the other variables, which remains as a limitation of our statistical analysis. Next, univariate and multivariate logistic regression analyses were conducted separately to assess the prevalence of ROP discordance based on pairing (paired/unpaired) and the number of siblings (twins/triplets).

Stepwise Poisson regression analysis was employed to identify significant predictors of ROP discordance, modeling the incidence rate. This assessed the impact of variables like sex, BW, oxygen saturation, maternal age, and medical interventions on ROP variability. Potential interactions (e.g., GA × BW) were explored. A post-hoc power analysis evaluated the study’s ability (*n* = 88 discordant neonates) to detect meaningful associations given observed effect sizes, addressing whether non-significant findings might stem from limited sample size or low event rates. All analyses used a significance threshold of *p* < 0.05 (two-sided tests).

## Results

**Fig. 1:**
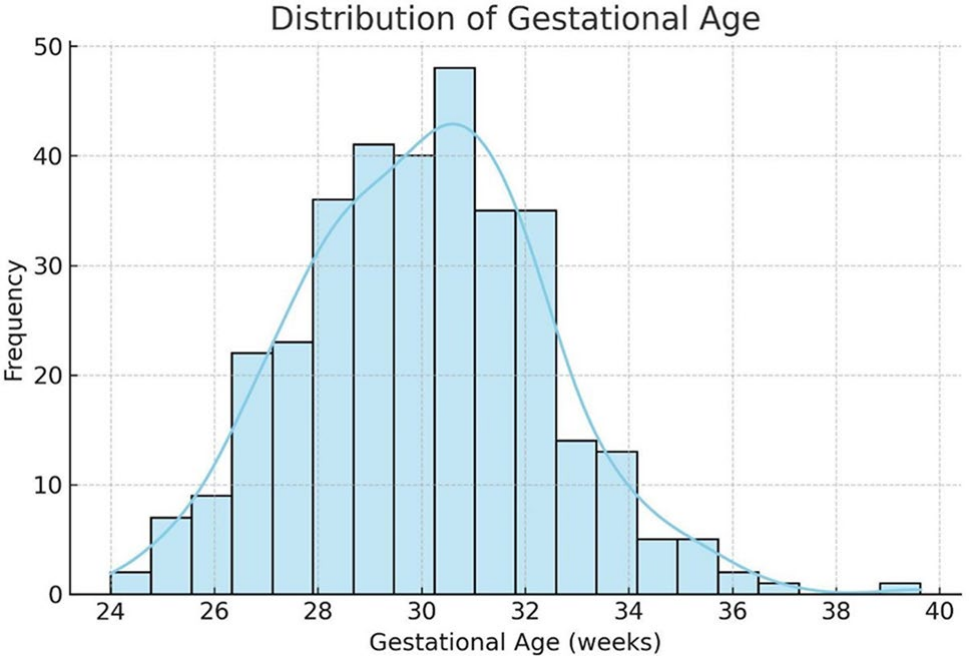
Distribution of gestational age among the study population indicates a non-normal, slightly right-skewed distribution. The overlaid kernel density estimate (KDE) highlights clustering around lower gestational age values

The study encompassed 339 neonates, comprising 168 twins (49.6%) and 171 triplets (50.4%). The severity of retinopathy varied, with 165 cases of mild retinopathy (48.7%), 164 cases of moderate retinopathy (48.4%), and 10 cases of severe retinopathy (2.9%). The subjects included 183 males (54.0%) and 156 females (46.0%) (Table 1). Normality tests analysis for gestational age showed a statistically significant deviation from normality (Shapiro-Wilk: W = 0.967, *p* < 0.001; Kolmogorov-Smirnov: D = 0.134, *p* < 0.001) (see Fig. 1).

**Table 1:** Descriptive characteristics of the study population (*n* = 339)

Variable	Frequency (%) / Mean (95% CI)
Qualitative Variables	
Multiple Gestation	
Twin	168 (49.6%)
Triplet	171 (50.4%)
Laser Treatment	
Not done	329 (97.1%)
Done	10 (2.9%)
Intravitreal Bevacizumab (IVB) Treatment	
Not done	337 (99.7%)
Done	1 (0.3%)
Missing	1 (0.3%)
Sex	
Male	183 (54.0%)
Female	156 (46.0%)
Number of Blood Transfusion(s)	
0	296 (87.3%)
1	36 (10.6%)
2	4 (1.2%)
3	3 (0.9%)
Sepsis	
No	235 (98.8%)
Yes	4 (1.2%)
Systemic Disease***	
No	313 (92.3%)
Yes	26 (7.7%)
Method of Delivery	
Cesarean Delivery	335 (98.8%)
Normal Vaginal Delivery (NVD)	4 (1.2%)
ROP Grade	
Mild	165 (48.7%)
Moderate	164 (48.4%)
Severe	10 (2.9%)
Quantitative Variables	**Mean (95% Confidence Interval)**
Gestational Age (weeks)	32.84 (32.66–33.02)
Birth Weight (grams)	1747.74 (1709.28–1786.20)
Duration of Oxygen Therapy (days)	5.84 (4.92–6.76)
NICU Stay Duration (days)	13.62 (12.52–14.72)
Duration of Mechanical Ventilation (days)	3.77 (2.57–4.96)
Maternal Age (years)	29.62 (28.98–30.26)

***Systematic disease was considered to be any neonatal comorbidities, including the following categories: pulmonary, cardiovascular, neurological, gastrointestinal, thyroid, renal, hematologic, and endocrine disorders

Discordance in severity of ROP (stage difference between siblings) was observed in 88 multiple birth neonates (26.0%). Among the 88 neonates with discordant ROP severity, 52 were twins (59.09%) and 36 were triplets (40.09%). Laser treatment was performed in 6 infants (6.82%), while 82 infants (93.18%) did not receive it. Intravitreal Bevacizumab (IVB) treatment was administered to 1 infant (1.14%). The male-to-female ratio was 48:40. Most infants (84, 95.45%) did not require blood transfusions. Sepsis occurred in 1 case (1.14%), and 7 infants (7.95%) had systemic diseases (any neonatal comorbidities, including the following categories: pulmonary, cardiovascular, neurological, gastrointestinal, thyroid, renal, hematologic, and endocrine disorders).

Cesarean delivery was the predominant method of delivery (86 infants, 97.73%). The mean GA was 33.21 weeks (± 1.88), BW averaged 1810 g (± 404.60), and NICU duration was 10.94 days (± 9.79). Ventilation duration was 0.35 days ± 1.35, and maternal age averaged 29.76 years (± 5.67). Of the discordant infants, 46.59% were diagnosed with mild ROP, 45.45% with moderate ROP, and 7.95% with severe ROP requiring treatment (Tables 2.1 and 2.2).

**Table 2.1:** Qualitative characteristics of twins and triplets with discordant ROP severity (*n* = 88)

Qualitative Variables	Category	Frequency (Percentage)
Number	Twin	52 (59.09%)
	Triplet	36 (40.91%)
	**Total**	**88 (100.0%)**
Laser Treatment	Not done	82 (93.18%)
	Done	6 (6.82%)
IVB Treatment	Not done	87 (98.86%)
	Done	1 (1.14%)
Sex	Male	48 (54.55%)
	Female	40 (45.45%)
Number of Blood Transfusion(s)	0	84 (95.45%)
	1	4 (4.55%)
	2	0 (0.00%)
	3	0 (0.00%)
Sepsis	No	87 (98.86%)
	Yes	1 (1.14%)
Systemic Disease	No	81 (92.05%)
	Yes	7 (7.95%)
Method of Delivery	Cesarean Delivery	86 (97.73%)
	NVD	2 (2.27%)
Discordance Grade	Mild	41 (46.59%)
	Moderate	40 (45.45%)
	Severe	7 (7.95%)

**Table 2.2:** Quantitative characteristics of twins and triplets with discordant ROP severity (*n* = 88)

Quantitative Variables	Mean ± Standard Deviation
Gestational Age (weeks)	33.21 ± 1.88
Birth Weight (grams)	1810 ± 404.60
O₂ Duration (days)	2.60 ± 5.59
NICU Stay Duration (days)	10.94 ± 9.79
Ventilation Duration (days)	0.35 ± 1.35
Maternal Age (years)	29.76 ± 5.67

In the univariate logistic regression analysis, none of the examined variables were significantly associated with the outcome. BW exhibited an odds ratio (OR) of 1.000 (95% CI: 0.999–1.001; *p* = 0.981). The sex of the subjects (male vs. female) had an OR of 1.069 (95% CI: 0.461–2.480; *p* = 0.876), and the duration of oxygen (O₂) therapy showed an OR of 1.055 (95% CI: 0.963–1.157; *p* = 0.248). The length of stay in the neonatal intensive care unit (NICU) resulted in an OR of 1.011 (95% CI: 0.968–1.056; *p* = 0.619), while the duration of mechanical ventilation had an OR of 1.337 (95% CI: 0.822–2.174; *p* = 0.241).

Blood transfusion frequency exhibited a higher odds ratio (OR) of 2.727; however, it was accompanied by a wide confidence interval (95% CI: 0.273–27.293; *p* = 0.393), indicating a lack of precision. The presence of sepsis resulted in an OR of 0.000, with an undefined upper confidence limit and a p-value of 1.000, suggesting complete data separation and hindering meaningful interpretation. Systemic disease (yes vs. no) demonstrated an OR of 1.178 (95% CI: 0.248–5.603; *p* = 0.837), while maternal age had an OR of 1.018 (95% CI: 0.945–1.097; *p* = 0.644). Finally, the route of delivery (normal vaginal delivery vs. cesarean section) presented an OR of 0.870 (95% CI: 0.053–14.357; *p* = 0.922) (Table 3).

**Table 3:** Summary of univariate logistic regression results for ROP

Variable	Odds Ratio	95% CI (Lower)	95% CI (Upper)	*P*-value
Birth Weight	1.000	0.999	1.001	0.981
Sex (male vs. female)	1.069	0.461	2.480	0.876
O2 Duration*	1.055	0.963	1.157	0.248
Duration of NICU	1.011	0.968	1.056	0.619
Ventilation Duration	1.337	0.822	2.174	0.241
Blood Transfusion Frequency**	2.727	0.273	27.293	0.393
Sepsis (yes vs. no)	0.000*	0.000	-	1.000
Systemic Disease*** (yes vs. no)	1.178	0.248	5.603	0.837
Maternal Age	1.018	0.945	1.097	0.644
Route of Delivery (NVD vs. C/S)	0.870	0.053	14.357	0.922

*O2 duration refers to the total number of days during which our patients received oxygen via various routes while extubated. **Blood transfusion frequency refers to the number of times patients require a blood transfusion. ***Systematic disease was considered to be any neonatal comorbidities, including the following categories: pulmonary, cardiovascular, neurological, gastrointestinal, thyroid, renal, hematologic, and endocrine disorders

**Fig. 2:**
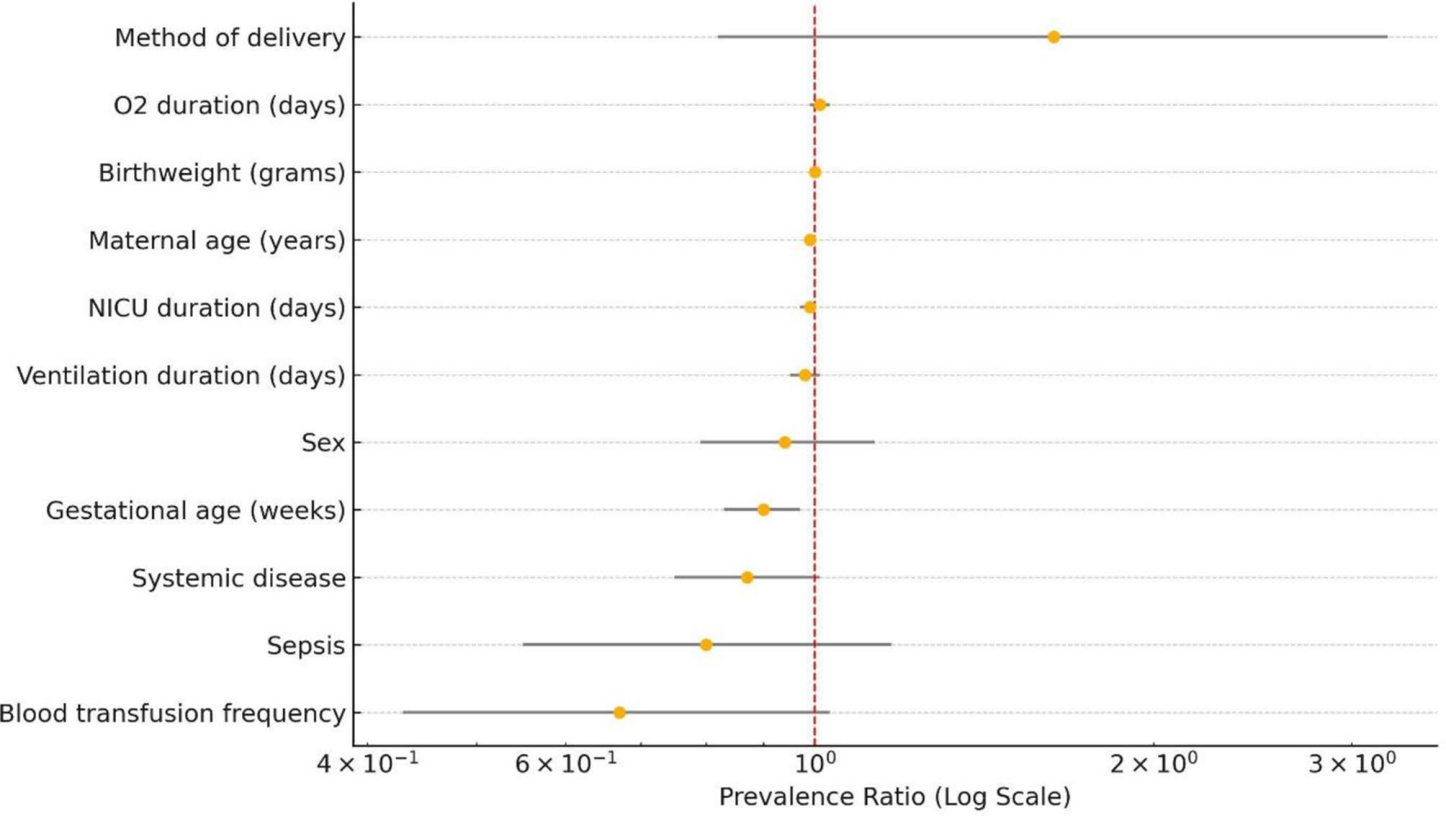
This figure displays the prevalence ratios (PR) with 95% confidence intervals for variables potentially influencing Retinopathy of Prematurity (ROP) in neonates with discordant ROP (*n* = 88), based on Poisson regression analysis. Each variable is plotted along the y-axis, with its corresponding PR and CI shown on a logarithmic x-axis. A red dashed vertical line at PR = 1.0 indicates the threshold for no association. Variables with CIs that do not cross 1 suggest a statistically significant association, such as gestational age. The figure highlights gestational age as a significant protective factor for ROP development

Multivariate logistic regression analysis of risk factors between siblings with less and more severe ROP in discordant pairs revealed no significant associations. Sex (*p* = 0.448), sepsis (*p* = 1.000), systemic disease (*p* = 1.000), method of delivery (*p* = 0.154), blood transfusion frequency (*p* = 0.552), GA (*p* = 0.075), birth weight (*p* = 0.562), O2 duration (*p* = 0.822), NICU duration (*p* = 0.614), ventilation duration (*p* = 0.893), and maternal age (*p* = 0.613) showed no statistically significant differences (Table 2.1, 2.2). Stepwise Poisson regression analysis identified GA as the only factor significantly associated with ROP severity (prevalence ratio = 0.90, 95% CI: 0.83–0.97, *p* = 0.012), while other variables, including sex (PR = 0.94, *p* = 0.541), blood transfusion frequency (PR = 0.67, *p* = 0.073), and birth weight (PR = 1.00, *p* = 0.602), showed no significant association with ROP discordance (Table 4; Fig. 2).

**Table 4:** Multivariate logistic regression analysis of risk factors for ROP in moderate and severe discordant neonates with ROP

Risk factors	Siblings with Moderate ROP	Siblings with Severe ROP	*P* value
Qualitative factors	**Number (Percentage (%))**	**Number (Percentage (%))**	
**Sex**			0.448
Male	43 (89.58%)	5 (10.42%)	
Female	38 (95.00%)	2 (5.00%)	
**Sepsis**			1.000
No	80 (91.95%)	7 (8.05%)	
Yes	1 (100.00%)	0 (0.00%)	
**Systemic disease**			1.000
No	74 (91.36%)	7 (8.64%)	
Yes	7 (100.00%)	0 (0.00%)	
**Method of delivery**			0.154
Cesarean-Delivery	80 (93.02%)	6 (6.98%)	
NVD	1 (50.00%)	1 (50.00%)	
**Quantitative factors**	Mean ± SD	Mean ± SD	
Blood transfusion frequency	0.04 ± 0.21	0 ± 0	0.552
Gestational age (weeks)	33.32 ± 1.90	32.00 ± 1.00	0.075
Birth weight (grams)	1817.40 ± 395.40	1724.28 ± 528.95	0.562
O₂ duration (days)	2.64 ± 5.78	2.14 ± 2.60	0.822
NICU duration (days)	11.09 ± 9.98	9.14 ± 7.60	0.614
Ventilation duration (days)	0.35 ± 1.40	0.28 ± 0.48	0.893
Maternal age (years)	29.85 ± 5.75	28.71 ± 4.82	0.613

**Fig. 3:**
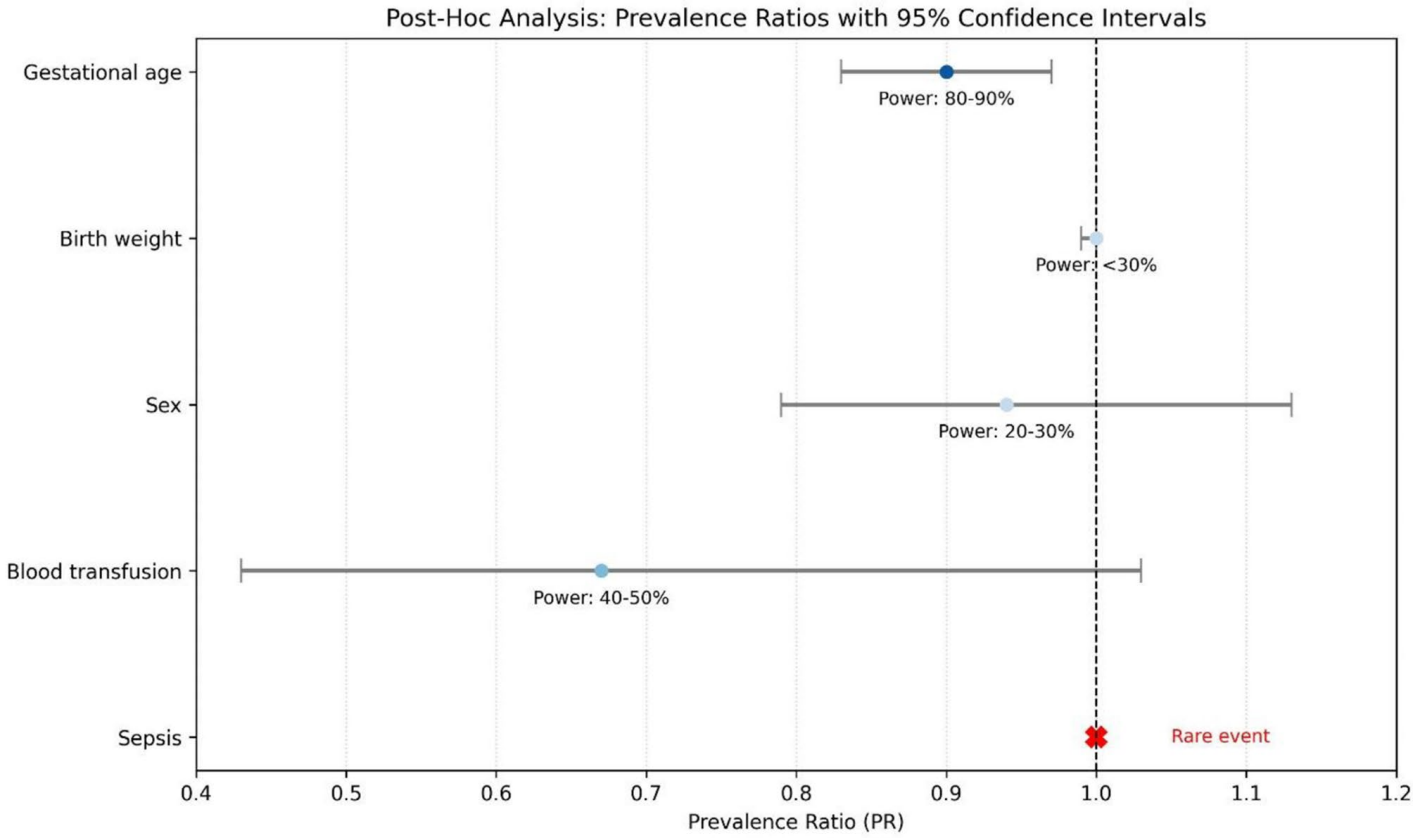
This figure presents post-hoc analysis of prevalence ratios (PR) with 95% confidence intervals for selected variables potentially associated with ROP. The vertical dashed line at PR = 1.0 represents the null value indicating no effect. Variables such as gestational age demonstrate higher statistical power (80–90%) and show a protective trend, while others like birth weight and sex show lower power (< 30% and 20–30%, respectively), indicating reduced reliability in detecting an effect. Blood transfusion has moderate power (40–50%) with wide confidence intervals, suggesting imprecision. Sepsis is marked separately as a rare event, reflecting its limited representation in the data

To further explore significant predictors of ROP discordance among twins and triplets, a post-hoc power analysis was conducted (Fig. 3). This analysis assessed the study’s ability to detect meaningful associations given the sample size of 88 discordant neonates and the observed effect sizes from the Poisson regression. The baseline discordance rate was 26.0% (88/339 neonates). For GA, with a prevalence ratio of 0.90 (95% CI: 0.83–0.97, *p* = 0.012), the study had adequate power (approximately 80–90%) to detect this moderate effect on ROP severity. However, for variables with small effect sizes, such as birth weight (PR = 1.00, 95% CI: 0.99-1.00, *p* = 0.602) and sex (PR = 0.94, 95% CI: 0.79–1.13, *p* = 0.541), power was low (approximately 20–30%), indicating the study was underpowered to detect subtle effects. For rare exposures, such as blood transfusion (PR = 0.67, 95% CI: 0.43–1.03, *p* = 0.073, with only 4.55% receiving transfusions) and sepsis (1.14% prevalence), power was also limited (40–50% or less), reflecting insufficient events to assess their impact. These findings suggest that the lack of significant predictors for ROP discordance may be due to limited statistical power, particularly for small effects or infrequent risk factors, rather than the absence of true associations.

## Discussion

ROP remains a leading cause of preventable childhood blindness, with a global pooled prevalence of 31.9% among preterm infants and 7.5% for severe cases requiring treatment [7]. Multiple births, such as twins and triplets, present a unique challenge due to the potential for discordant ROP severity among siblings, despite shared gestational environments.

Our cross-sectional study, conducted at the Poostchi ROP Clinic in southern Iran, investigated ROP discordance in 339 preterm infants (168 twins and 171 triplets). We observed a 26.0% prevalence of discordance, which was defined as a difference in ROP stage between siblings identified at any screening visit. The first ophthalmological examination was performed at 31 weeks postmenstrual age or 4 weeks after birth, whichever occurred later, in accordance with established screening guidelines. Subsequent examinations were scheduled based on the initial findings, with follow-up intervals determined by the stage and zone of ROP, following the ICROP recommendations and national screening protocols [5, 11].

Despite comprehensive analysis using stepwise Poisson regression, no significant predictors of discordance were identified, except for GA, which was significantly associated with ROP severity (prevalence ratio = 0.90, 95% CI: 0.83–0.97, *p* = 0.012). These findings highlight the complexity of ROP discordance and the urgent need to identify its predictors to enhance neonatal care.

Understanding ROP discordance is critical, particularly in developing countries where neonatal care resources are often limited [16–18]. Discordance complicates standard screening protocols, as siblings may require different levels of monitoring or treatment intensity despite having similar gestational profiles. For example, in resource-constrained settings such as certain regions of Iran, early identification of high-risk infants can optimize the allocation of limited ophthalmologic resources, thereby reducing the risk of blindness [8]. However, the existing literature reveals a significant gap in identifying specific risk factors for ROP discordance in multiple births, with most studies concentrating on overall ROP prevalence or severity rather than sibling differences [9]. Our study addresses this gap by examining discordance within a large cohort, providing insights that could inform tailored screening strategies to enhance outcomes in vulnerable populations.

The novelty of our study lies in its inclusion of both twins and triplets, a rare combination in ROP research, and its substantial sample size (*n* = 339), which enhances the robustness of our findings. Unlike most prior studies that focus solely on twins, our inclusion of 171 triplets provides a broader perspective on ROP discordance across various birth types. Conducted in southern Iran, the study also reflects a unique demographic and healthcare context, which may differ from high-income settings where most ROP research is concentrated. This diversity strengthens the generalizability of our findings and underscores the need for region-specific data to inform global ROP management strategies.

Our findings both align with and diverge from prior studies, reflecting the complexity of ROP discordance. Consistent with Shemesh et al. (2024), who studied very low BW twins, we identified GA as a significant factor in ROP severity [9]. However, unlike Campbell et al. (2025), who found that smaller twins in discordant pairs had a higher ROP risk after adjusting for BW and GA, our study did not identify BW as a predictor of discordance [10]. This discrepancy may stem from our inclusion of triplets, which may introduce different risk dynamics, or population-specific factors in Iran. Similarly, Zloto et al. (2020) and Petricli et al. (2019) reported that lower BW was associated with more severe ROP in discordant twins, a finding not replicated in our cohort [5, 11]. In contrast, Jin et al. (2022) found that smaller twins without significant BW discordance were not at increased risk for requiring ROP treatment, aligning with our observation that BW was not a significant predictor [12]. These variations suggest that ROP discordance is influenced by a combination of factors, including population demographics and study design, necessitating further investigation. In conclusion, while GA consistently predicts ROP severity, the role of BW in discordance remains controversial, highlighting the need for studies that account for diverse multiple birth types and regional contexts.

To address the gaps in our study, future research should focus on identifying individual risk factors for ROP discordance in twins and triplets. Multicenter studies with larger sample sizes are necessary to enhance statistical power and detect subtle or rare associations. Incorporating genetic profiling techniques, such as whole-exome sequencing or targeted gene panels, could help identify variants linked to ROP susceptibility [13]. Assessments of chorionicity, conducted through prenatal ultrasound or postnatal placental examination, would clarify the role of placental sharing in discordant outcomes [14]. Although our study did not directly assess genetic or prenatal factors, we briefly mentioned them to acknowledge potential underlying contributors to ROP discordance that may extend beyond the perinatal and clinical variables we analyzed.

Additionally, collecting data on prenatal exposures, such as maternal nutrition, infections, and assisted reproductive technologies, could reveal environmental influences on the variability of retinopathy of prematurity (ROP) [15]. Longitudinal designs could further elucidate temporal changes in ROP severity, providing insights into causality. By addressing these gaps, future studies can develop predictive models for ROP discordance, enabling personalized screening and intervention strategies to improve visual outcomes in preterm infants from multiple births.

### Study strengths

This research represents one of the few investigations that specifically evaluate the discordance of ROP stages in multiple-birth infants, such as twins and triplets, within the context of a middle-income developing country. The study contributes to the existing literature by analyzing postpartum clinical and demographic risk factors while employing a structured classification system based on the ICROP to quantify the severity of discordance. It utilizes a relatively large sample of preterm infants from multiple births, adheres to standardized screening protocols, and classifies ROP discordance according to internationally recognized criteria (ICROP).

### Study limitations

This study on ROP discordance in multiple births is subject to limitations that may have influenced its findings. A post-hoc power analysis revealed that while the study was adequately powered (80–90%) to detect the association between GA and ROP severity (prevalence ratio = 0.90, *p* = 0.012), it was underpowered (< 50%) for detecting predictors of discordance with small effect sizes (e.g., BW, sex) or low-prevalence events (e.g., sepsis, blood transfusion). This suggests that the absence of significant predictors may reflect insufficient statistical power rather than a lack of true associations, necessitating larger, multicenter studies to explore these relationships more robustly.

Additionally, the study lacked data on critical biological and environmental factors that could contribute to ROP discordance. Genetic screening was not performed, limiting the ability to identify heritable factors influencing ROP severity differences among siblings. Similarly, data on chorionicity and placenta sharing, which can lead to unequal resource distribution in multiple pregnancies, were not collected, potentially missing key determinants of discordance. Prenatal environmental exposures, such as maternal nutrition or infections, were not included in our analysis, as the study focused on identifying postpartum risk factors contributing to ROP discordance in twins and triplets, despite the recognized potential of prenatal factors to influence fetal development differentially. These gaps restrict a comprehensive understanding of ROP discordance, underscoring the need for future research to incorporate these variables to enhance neonatal care strategies.

## Conclusion

Our study underscores the prevalence and complexity of ROP discordance in multiple births, with a 26.0% discordance rate and GA as a key factor in ROP severity. The absence of significant predictors for discordance highlights the multifactorial nature of this phenomenon and the limitations of our study, including the lack of genetic, chorionicity, and prenatal exposure data. While our study did not directly assess genetic or prenatal factors, we briefly mentioned them to acknowledge possible underlying contributors to ROP discordance that may extend beyond the perinatal and clinical variables we analyzed. By building on these findings, future research can bridge these gaps, paving the way for tailored neonatal care strategies that reduce the burden of ROP-related blindness, particularly in resource-limited settings.

## Data Availability

Data is provided within the manuscript.

## References

[1] Blencowe H, Lawn JE, Vazquez T, Fielder A, Gilbert C. Preterm-associated visual impairment and estimates of retinopathy of prematurity at regional and global levels for 2010. Pediatr Res. 2013;74(Suppl 1):35–49. PMID: 24366462; PMCID: PMC3873709. 24366462 10.1038/pr.2013.205 PMC3873709

[2] Athikarisamy SE, Vinekar A, Patole S. Retinopathy of prematurity in India - what can we learn from the polio legacy? Lancet Reg Health Southeast Asia. 2023;14:100210. 10.1016/j.lansea.2023.100210. PMID: 37492414; PMCID: PMC10363497. 37492414 10.1016/j.lansea.2023.100210 PMC10363497

[3] Riazi-Esfahani M, Alizadeh Y, Karkhaneh R, Mansouri MR, Kadivar M, Nili Ahmadabadi M, Nayeri F. Retinopathy of prematurity: single versus Multiple-Birth pregnancies. J Ophthalmic Vis Res. 2008;3(1):47–51. PMID: 23479522; PMCID: PMC3589219. 23479522 PMC3589219

[4] Dos Santos Motta MM, Fortes Filho JB, Coblentz J, Fiorot CA. Multiple pregnancies and its relationship with the development of retinopathy of prematurity (ROP). Clin Ophthalmol. 2011;5:1783–7. 10.2147/OPTH.S25431. Epub 2011 Dec 20. PMID: 22267912; PMCID: PMC3258087. 22267912 10.2147/OPTH.S25431 PMC3258087

[5] Petricli İS, Kara C, Işık DU, Demirel N, Baş AY. Effect of birth weight on retinopathy of prematurity in discordant twin pairs. Indian J Ophthalmol. 2019;67(6):806–10. 10.4103/ijo.IJO_1197_17. PMID: 31124491; PMCID: PMC6552611. 31124491 10.4103/ijo.IJO_1197_17 PMC6552611

[6] Şekeroğlu MA, Hekimoğlu E, Çelik Ü, Kale Y, Baş AY. Retinopathy of prematurity in triplets. Turk J Ophthalmol. 2016;46(3):114–7. 10.4274/tjo.94815. Epub 2016 Jun 6. PMID: 27800273; PMCID: PMC5076293. 27800273 10.4274/tjo.94815 PMC5076293

[7] Blazon MN, Rezar-Dreindl S, Wassermann L, Neumayer T, Berger A, Stifter E. Retinopathy of prematurity: incidence, risk factors, and treatment outcomes in a tertiary care center. J Clin Med. 2024;13(22):6926. 10.3390/jcm13226926. PMID: 39598070; PMCID: PMC11594805. 39598070 10.3390/jcm13226926 PMC11594805

[8] Le TP, Feng J, Ding L, Hu R, Lou XB, Ulrich JN, Cabrera MT. Survey of current retinopathy of prematurity practices in China. Int J Ophthalmol. 2021;14(8):1241–7. PMID: 34414091; PMCID: PMC8342299. 34414091 10.18240/ijo.2021.08.17 PMC8342299

[9] Shemesh R, Strauss T, Zaslavsky-Paltiel I, Lerner-Geva L, Reichman B, Wygnanski-Jaffe T. Israel neonatal network. Perinatal and neonatal risk factors for retinopathy of prematurity in very low birthweight, very preterm twins: a population-based study. Eye (Lond). 2024;38(5):902–9. 10.1038/s41433-023-02801-8. Epub 2023 Nov 4. PMID: 37925560; PMCID: PMC10965998. 37925560 10.1038/s41433-023-02801-8 PMC10965998

[10] Campbell MA, Grove NC, McDuffie RS Jr, Auer EA, McReynolds AJ, McCourt EA, Wymore EM, Mandava N, Wagner BD, Mathias MT, Oliver SCN, Jung JL, Lynch AM. The relationship between discordant birth weight in twin pairs and the development of retinopathy of prematurity. J AAPOS. 2025;29(1):104095. Epub 2024 Dec 31. PMID: 39746539. 39746539 10.1016/j.jaapos.2024.104095

[11] Zloto O, Goldfinger Lerner M, Mazkereth R, Spierer A, Yinon Y. Retinopathy of prematurity in discordant twins: is the small twin at increased risk? Graefes Arch Clin Exp Ophthalmol. 2020;258(4):893–8. 10.1007/s00417-019-04597-4. Epub 2020 Jan 8. PMID: 31915974. 31915974 10.1007/s00417-019-04597-4

[12] Jin E, Wang Z, Yao L, Yin H, Zhao M. Treatment for retinopathy of prematurity in twins: the small twin without high birth weight discordant is not at increased risk. Child (Basel). 2022;9(6):891. 10.3390/children9060891. PMID: 35740828; PMCID: PMC9222126. 10.3390/children9060891 PMC9222126 35740828

[13] Ortega-Molina JM, Anaya-Alaminos R, Uberos-Fernández J, Solans-Pérez de Larraya A, Chaves-Samaniego MJ, Salgado-Miranda A, Piñar-Molina R, Jerez-Calero A, García-Serrano JL. Genetic and environmental influences on retinopathy of prematurity. Mediators Inflamm. 2015;2015:764159. 10.1155/2015/764159. Epub 2015 May 21. PMID: 26089603; PMCID: PMC4454750. 26089603 10.1155/2015/764159 PMC4454750

[14] Yau GSK, Lee JWY, Tam VTY, Yip S, Cheng E, Liu CCL, Chu BCY, Wong IYH. Incidence and risk factors for retinopathy of prematurity in multiple gestations: a Chinese population study. Med (Baltim). 2015;94(18):e867. PMID: 25950699; PMCID: PMC4602518. 10.1097/MD.0000000000000867 PMC4602518 25950699

[15] Di Pietro M, Decembrino N, Afflitto MG, Malerba E, Avitabile T, Franco LM, Longo A, Betta P. Risk factors in the development of retinopathy of prematurity: A 10-year retrospective study. Early Hum Dev. 2023;185:105844. 10.1016/j.earlhumdev.2023.105844. Epub 2023 Aug 19. PMID: 37672895. 37672895 10.1016/j.earlhumdev.2023.105844

[16] Uberos J, Fernández-Marin E, Campos-Martínez A, Ruiz-López A, García-Serrano JL. Analysis of the association between in vitro fertilization/assisted conception and the development of retinopathy of prematurity in Very-Low-Birth weight newborns. Turk Arch Pediatr. 2024;59(6):547–52. PMID: 39540751; PMCID: PMC11562287. 39540751 10.5152/TurkArchPediatr.2024.24157 PMC11562287

[17] Farajizade S, Karimi MS. The shocks of climate change on economic growth in developing countries: evidence from a panel smooth transition regression model. J Clean Prod. 2022;368:133151. 10.1016/j.jclepro.2022.133151.

[18] Tadayon AMD, Hosseini HMD, Yousefi AMD, Abasi. Pariya MDb; Fatehi, Negar MDc; Yousufzai, Shayan MDb,*. Innovative use of French tubes in pediatric choanal atresia surgery– affordable solutions for resource-limited areas: a retrospective descriptive study. International Journal of Surgery Open. 2025;63(3):194–198. 10.1097/IO9.0000000000000273

